# Longitudinal evaluation of structural brain alterations in two established mouse models of Gulf War Illness

**DOI:** 10.3389/fnins.2024.1465701

**Published:** 2024-09-06

**Authors:** Jessica M. Carpenter, Sarah N. Hughes, Nikolay M. Filipov

**Affiliations:** Department of Physiology and Pharmacology, College of Veterinary Medicine, University of Georgia, Athens, GA, United States

**Keywords:** Gulf War Illness, magnetic resonance imaging (MRI), pyridostigmine bromide, permethrin, DEET, diisopropylfluorophosphate (DFP), corticosterone, longitudinal

## Abstract

Gulf War Illness (GWI) affects nearly 30% of veterans from the 1990–1991 Gulf War (GW) and is a multi-symptom illness with many neurological effects attributed to in-theater wartime chemical overexposures. Brain-focused studies have revealed persistent structural and functional alterations in veterans with GWI, including reduced volumes, connectivity, and signaling that correlate with poor cognitive and motor performance. GWI symptomology components have been recapitulated in rodent models as behavioral, neurochemical, and neuroinflammatory aberrations. However, preclinical structural imaging studies remain limited. This study aimed to characterize the progression of brain structural alterations over the course of 12 months in two established preclinical models of GWI. In the PB/PM model, male C57BL/6 J mice (8–9 weeks) received daily exposure to the nerve agent prophylactic pyridostigmine bromide (PB) and the pyrethroid insecticide permethrin (PM) for 10 days. In the PB/DEET/CORT/DFP model, mice received daily exposure to PB and the insect repellent DEET (days 1–14) and corticosterone (CORT; days 7–14). On day 15, mice received a single injection of the sarin surrogate diisopropylfluorophosphate (DFP). Using a Varian 7 T Bore MRI System, structural (sagittal T2-weighted) scans were performed at 6-, 9-, and 12-months post GWI exposures. Regions of interest, including total brain, ventricles, cortex, hippocampus, cerebellum, and brainstem were delineated in the open source Aedes Toolbox in MATLAB, followed by brain volumetric and cortical thickness analyses in ImageJ. Limited behavioral testing 1 month after the last MRI was also performed. The results of this study compare similarities and distinctions between these exposure paradigms and aid in the understanding of GWI pathogenesis. Major similarities among the models include relative ventricular enlargement and reductions in hippocampal volumes with age. Key differences in the PB/DEET/CORT/DFP model included reduced brainstem volumes and an early and persistent loss of total brain volume, while the PB/PM model produced reductions in cortical thickness with age. Behaviorally, at 13 months, motor function was largely preserved in both models. However, the GWI mice in the PB/DEET/CORT/DFP model exhibited an elevation in anxiety-like behavior.

## 1 Introduction

Gulf War Illness (GWI), a persisting multi-symptom malady predominantly affecting the nervous and immune systems, presents in about a third of the veterans from the 1990–1991 Gulf War (White et al., 2016). Major symptoms include fatigue, aberrations in cognition/memory, reduced motor function, and mood disturbances. Precise etiology of GWI is still unknown but it is largely attributed to war-time chemical overexposures including to insecticides/repellents [permethrin (PM), chlorpyrifos, DEET], nerve agents (sarin/cyclosarin), and a nerve agent prophylactic (pyridostigmine bromide; PB). While the understanding of GWI has improved, symptoms remain heterogenous among affected veterans and curative treatments do not exist; however, therapeutic interventions are being explored (White et al., 2016).

Several clinical imaging studies have evaluated the neuroanatomical correlates to the progressive, long-term aberrations in GWI symptomology. These structural magnetic resonance imaging (MRI) studies found specific reductions in white and gray matter volumes in multiple cortical and subcortical areas (e.g., hippocampus, hypothalamus, brainstem, cerebellum) in GWI veterans (Chao et al., 2011; Rayhan et al., 2013; Chao et al., 2015; Christova et al., 2017; James et al., 2017; Van Riper et al., 2017; Chao, 2020; Zhang et al., 2020; Zhang et al., 2021). Functional MRI (fMRI) studies corroborated these structural abnormalities by detecting significant signal changes (e.g., activity) in regions governing cognitive, memory, motor and mood function such as the prefrontal, somatosensory, and motor cortices (Gopinath et al., 2012; Wylie et al., 2019), as well as the thalamus, caudate, and hippocampus (Calley et al., 2010; Li et al., 2011; Cooper et al., 2016). Across these studies, the patient populations included veterans diagnosed with GWI based on the Haley criteria which groups GWI symptoms into three syndromes: (1) impaired cognition (i.e., problems attention, memory, reasoning, insomnia, depression, daytime sleepiness, and headaches); (2) confusion-ataxia (i.e., problems thinking, disorientation, balance, vertigo, and impotence); and (3) arthromyoneuropathy (i.e., joint and muscle pain, muscle fatigue, difficulty lifting, and extremity paresthesia) (Haley et al., 1997; IOM, 2014). These studies found marked behavior aberrations that correlated with functional abnormalities (Calley et al., 2010; Li et al., 2011; Gopinath et al., 2012; Cooper et al., 2016; Wylie et al., 2019). Additionally, an earlier clinical study detected neuronal loss in the basal ganglia with corresponding dopamine dysfunction (Haley et al., 2000). The structural and functional abnormalities observed in these areas are consistent with reported symptomology by veterans and correlate strongly with performance deficits in working memory, attention, and motor tasks as well as with mood impairments, pain, and sleep quality (Calley et al., 2010; Hubbard et al., 2014; Clarke et al., 2019; Chao, 2020; Washington et al., 2020; Zhang et al., 2021). However, neurological abnormalities have not been correlated with types and/or severity of GWI exposures.

Many of the symptoms exhibited by GWI veterans have been recapitulated in GWI rodent models. Studies utilizing these models have demonstrated multiple behavioral deficits (i.e., cognition/memory, motor, and mood) and biological parameters (i.e., neuroinflammation) consistent with symptomology in GWI veterans (Parihar et al., 2013; Megahed et al., 2014; O’Callaghan et al., 2015; Zakirova et al., 2015; Zakirova et al., 2016; Miller et al., 2018; Macht et al., 2019; Dickey et al., 2021; Ribeiro and Deshpande, 2021). To date, our group has explored the acute and long-term neurobiological and behavioral alterations (Carpenter et al., 2020; Brown et al., 2021a; Brown et al., 2021b; Carpenter et al., 2021; Carpenter et al., 2022) in two established GWI models, one utilizing subacute PB and PM exposure (Zakirova et al., 2015; Zakirova et al., 2016) and another employing PB, DEET, corticosterone (CORT), and diisopropylfluorophosphate (DFP) exposure (O’Callaghan et al., 2015) In these studies, we observed: (1) acute monoaminergic disbalance in multiple brain regions associated with motor, memory, and mood function (Carpenter et al., 2020), (2) hippocampal neuroinflammation (Carpenter et al., 2020; Carpenter et al., 2021; Carpenter et al., 2022) and (3) changes in hippocampal synaptic plasticity and transmission (Brown et al., 2021a; Brown et al., 2021b). Further, our long-term studies (6–10 months post GWI exposures) revealed multiple GWI-related behavioral deficits in motor function, cognition, and mood (Carpenter et al., 2021; Carpenter et al., 2022) and alterations in hippocampal electrophysiology (Brown et al., 2021a; Carpenter et al., 2021).

While rodent GWI models produce GWI-like behavioral and pathological phenotypes, functional and structural imaging studies investigating the neuroanatomical changes within GWI models remain limited (Koo et al., 2018; Wu et al., 2021). This may be due to the complexity of conducting rodent imaging studies, especially studies that involve repeated imaging over time. Nevertheless, results from the few available GWI preclinical studies provide highly translational and valuable insights into this chronic illness (Koo et al., 2018; Wu et al., 2021). In particular, high order diffusion MRI revealed distinct cortical and subcortical (hippocampus and hypothalamus) alterations that corresponded to neuroinflammation following exposure to the GWI-relevant chemicals, DFP and CORT (Koo et al., 2018). Additionally, structurally significant increases in lateral ventricle volume and decreases in hippocampal and thalamic volumes were observed in another GWI model (PB, PM, DEET, and restraint stress) 10 months post exposure that aligned with neurobehavioral impairments in cognition and mood function (Wu et al., 2021).

Due to the paucity of longitudinal information surrounding GWI preclinical neuroanatomical changes and possible GWI exposure specific differences, the present study aimed to characterize the progression of neuroanatomical alterations over the course of 12 months following two, distinct GWI chemical exposure paradigms. Utilizing the PB/PM (Zakirova et al., 2015) and PB/DEET/CORT/DFP (O’Callaghan et al., 2015) models, we evaluated GWI neuroanatomical volume changes at 6-, 9-, and 12-months post exposure in various brain regions including the total brain, ventricles, cortex, hippocampus, cerebellum, and brainstem. Cortical thickness measurements were also conducted over the course of the study, alongside limited behavioral and stress responsivity evaluations at 13 months.

## 2 Materials and methods

### 2.1 Materials

The following chemicals were used for animal treatments: pyridostigmine bromide (PB; Sigma Aldrich, St. Louis, MO), permethrin (PM; 29.5% cis/69.5% trans isomer; Chem Service Inc., West Chester, PA), diisopropylfluorophosphate (DFP; Sigma Aldrich), N-Diethyl-3-methylbenzamide (DEET; Sigma Aldrich), and corticosterone (CORT; Steraloids, Newport, RI). All additional chemicals and reagents used in this study, unless otherwise noted, were of analytical or higher grade and were obtained from Sigma Aldrich or Fisher Scientific (Hampton, NH).

### 2.2 Animals and GWI models

#### 2.2.1 Animals

Male C57BL/6 J mice (8–9 weeks old; Jackson Laboratories, Bar Harbor, ME) were housed 4 per cage in an environmentally controlled room (22–24°C) and maintained on a 12 h light/dark cycle (0700–1900 lights on) throughout the study. Mice were handled daily for 1 week prior to the start of the study to minimize experimenter-induced stress. Food and water were available *ad libitum*. All procedures were approved in advance by the University of Georgia Institutional Animal Care and Use Committee (IACUC), as well as by DoD ACURO, and were in accordance with the latest National Institutes of Health guidelines.

Body weights were measured daily during the GWI chemicals exposure and then biweekly following the last GWI exposure until study completion. All mice were euthanized 13.5 months post GWI chemicals exposure (CO_2_, followed by decapitation). When the mice were euthanized, body weight and length were measured to determine morphometric-related parameters including body mass index [BMI; Body weight (g)/length^2^ (cm^2^)] and the Lee index [body weight (g)/length(cm)] as in Novelli et al. (2007). Brains were extracted, weighed, and split sagittally before one half was quickly frozen on dry ice and the other half was immersion fixed in 4% paraformaldehyde as in Carpenter et al. (2021) for future analyses.

#### 2.2.2 GWI models

**Figure 1:**
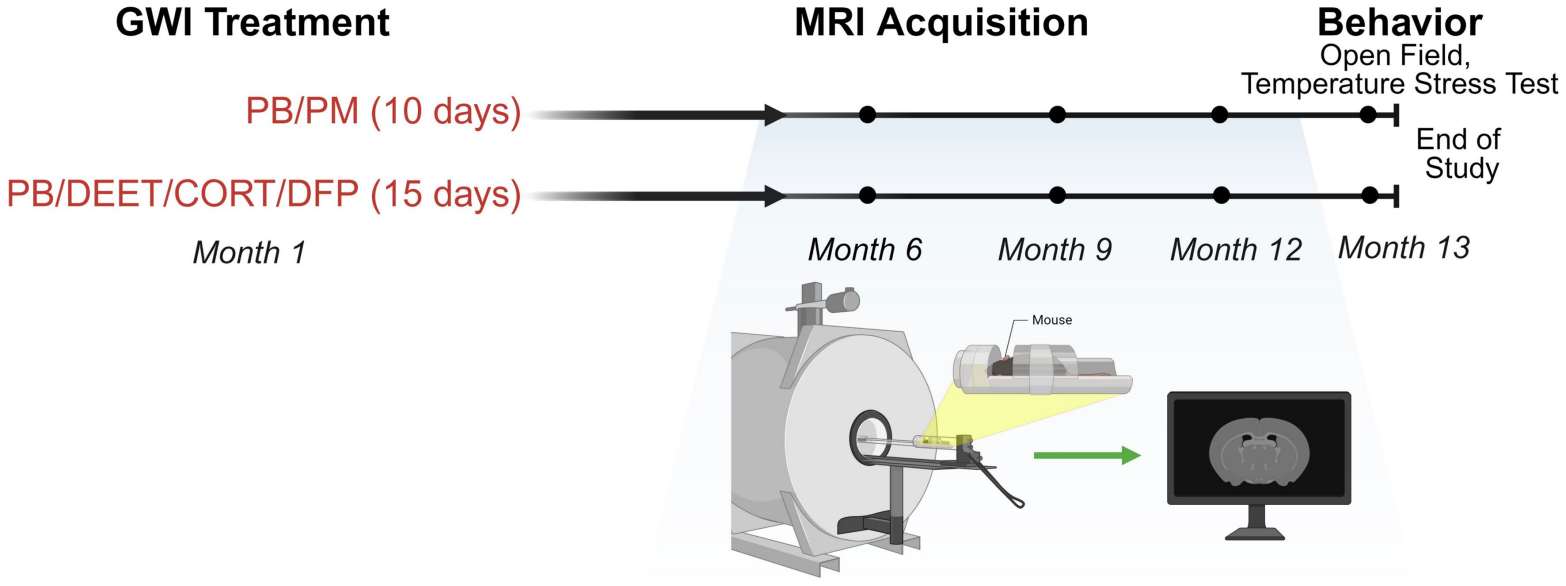
Experimental timeline and MRI collection. Experimental timeline for the study investigating two GWI exposure paradigms (PB/PM: 10 days; PB/DEET/CORT/DFP: 15 days). MRI evaluations were conducted at 6-, 9-, and 12-months post-exposure. Open field and temperature response stress tests were performed at 13 months post-exposure. Figure created with BioRender.com. CORT, corticosterone; DEET, N,N-diethyl-meta-toluamide; DFP, diisopropylfluorophosphate; GWI, Gulf War Illness; MRI, magnetic resonance imaging; PB, pyridostigmine bromide; PM, permethrin.

Two established, chemically different GWI treatment paradigms were utilized for this study (Figure 1). For both models, mice were selected for treatment randomly. Following the Zakirova et al. model (Zakirova et al., 2015), mice were co-administered the nerve agent prophylactic pyridostigmine bromide [PB; 0.7 mg/kg body weight (BW); IP] and the pyrethroid insecticide permethrin (PM; 200 mg/kg BW; IP) or DMSO vehicle over 10 days. Following the O’Callaghan et al. model (O’Callaghan et al., 2015), mice received daily administration of PB (2 mg/kg BW; SC) and the insect repellent DEET (30 mg/kg BW; SC) or saline control for 14 days with concurrent stress [corticosterone (CORT): 200 mg/kg in 1.2% EtOH drinking water] exposure on days 8–14. Control mice during this period (day 8–14) received 1.2% EtOH drinking water. On day 15, mice received a single dose of the nerve agent surrogate diisopropylfluorophosphate (DFP; 3.75 mg/kg BW; IP) or saline control. This resulted in 4 treatment groups (*N* = 24, *n* = 6/group/per model).

### 2.3 Structural magnetic resonance imaging

Structural brain images were acquired using a 7 Tesla magnet (Agilent, Santa Clara, CA), and MRI sequences were conducted on mice at 6, 9, and 12 months post GWI chemicals exposures (Figure 1). Within a model, order of imaging was random. For all imaging processing post collection, individual animals were coded, and MRI data were processed and analyzed by a treatment-blinded experimenter. Prior to imaging, mice were anesthetized with isoflurane (3% for induction, 1.0–1.5% for maintenance) in a 30%:70% O_2_:N_2_ gas mixture with a flow rate 0.8–1.0 L/min. Respiratory rate was monitored using a small animal monitoring system (Small Animal Instruments, Inc., Stony Brook, NY) throughout the imaging period. Axial, two dimensional (2D) T1 and T2 weighted images were obtained using a spin echo sequence with the following acquisition parameters: TR 500 ms, TE 17 ms, 8 averages, FOV 25, data matrix 256 × 256, 17 slices, thickness 1.00 mm with no gap (Krishna et al., 2014). Sagittal, 3D T2 weighted images were obtained using a fast spin echo sequence with the following parameters: TR 4,000 ms, TE 33.38 ms, 2 averages, FOV 35, data matrix 256 × 256, 15 slices, thickness 1.00 mm with no gap.

#### 2.3.1 Volume measurements

**Figure 2:**
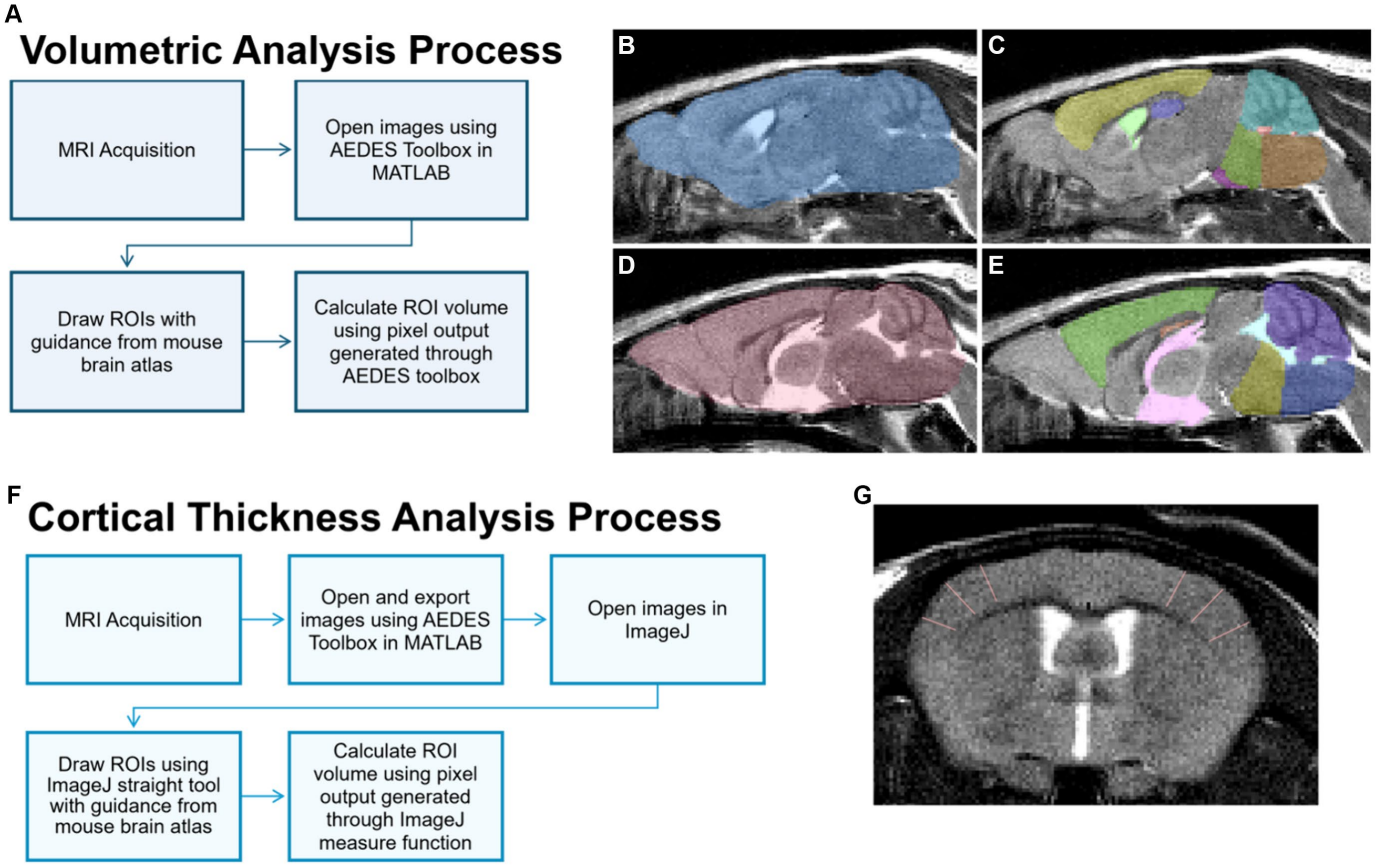
MRI image processing for volume and cortical thickness analyses. **(A)** For volume analysis of brain regions, sagittal T2 images were processed and brain regions of interest (ROIs) were delineated manually using the MATLAB Aedes toolbox. Representative ROIs depicted include the total brain **(B,D)**, cortex (**C**, yellow; **E**, green), hippocampus (**C**, purple; **E**, orange), cerebellum (**C**, teal; **E**, purple), medulla (**C**, orange; **E**, dark blue), pons (**C**, green; **E**, yellow), lateral ventricle (**C**, light green), 3rd ventricle (**E**, pink), and 4th ventricle (**C**, red; **E**, light blue). **(F)** For the cortical thickness analysis, cortical ROIs were delineated using the straight tool in ImageJ on T2-weighted axial images, as represented in **(G)** showing in the somatosensory cortex.

Images were displayed and masked using the open-source MATLAB toolbox AEDES using methods outlined in (Minkeviciene et al., 2019; Grant et al., 2020). Regions of interest (ROIs) including total brain, cortex, hippocampus, cerebellum, medulla, pons, and ventricles (i.e., lateral, 3^rd^, and 4^th^) were manually drawn according to the Allen Brain Atlas.^1^ ROIs were saved individually as masks, and volumetric analysis was conducted on the masks by using the pixel count generated with the AEDES toolbox. Volume (mm^3^) was calculated by first calculating the voxel volume $\left[\frac{FOV}{\mathrm{Matrix}}\times \frac{FOV}{\mathrm{Matrix}}\times \mathrm{Thickness}\right]$ and then multiplying the measured output with the calculated voxel volume. Refer to Figures 2A–E for volume analysis process and representative masks.

#### 2.3.2 Thickness measurements

Axial images were exported using the open-source MATLAB toolbox AEDES. Thickness measurements were done using the straight tool in ImageJ by manually drawing the thickness of ROIs including the frontal, motor, auditory, somatosensory, and dorsolateral entorhinal cortices as in Minkeviciene et al. (2019). Six measurement lines were drawn per slice, and line thickness in pixels (e.g., the number of pixels for cortical width) was obtained using the measure function in ImageJ. Cortical thickness was calculated by converting the number of pixels into mm. Refer to Figures 2F,G for the cortical thickness analysis process and a representative image.

### 2.4 Behavioral analyses

Limited behavioral assays were conducted at 13 months to evaluate sensitivity to a mild stressor, locomotor activity, and anxiety-like activity. The experimenter was treatment-blinded during the behavioral tests and behavioral sequence was randomized.

#### 2.4.1 Open field

Locomotor activity and anxiety-like behavior were assessed as previously described (Carpenter et al., 2021). Briefly, mice were individually placed into an open field arena (25 cm × 25 cm × 40 cm; Coulbourn Instruments, Whitehall, PA) and allowed to freely explore for 30 min. Locomotor parameters (i.e., distance traveled) and anxiety-like behaviors (i.e., entries and time spent in the center and corners) were scored using AnyMaze software (Stoelting) for the total 30 min and per 5 min intervals.

#### 2.4.2 Temperature response stress test

The temperature response stress test was used to assess stress-induced hyperthermia following methodology outlined by Borsini et al. (1989) and Bouwknecht et al. (2000). Rectal temperature measurements were obtained utilizing a thermocouple meter (Digi-Sense Single-Input thermometer) with a mouse-specific probe. Two temperature readings were recorded with a 15-min interval between each reading. After the first reading, the mouse was individually housed until the second reading. The change in temperature was calculated and analyzed.

### 2.5 Statistical analysis

All data were analyzed first by using preplanned comparisons within a model and a time point using Student’s *t-*test. To determine the effects of treatment over time or treatment x time interactions, a two-way repeated analysis of variance (RM-ANOVA) within a GWI model was used. Here, if a RM-ANOVA was significant (*p* ≤ 0.05), treatment means were separated by Student–Newman–Keuls (SNK) *post hoc* test. All data were analyzed using SigmaPlot 12.5 (San Jose, CA), and graphs were generated using GraphPad Prism (San Diego, CA).

## 3 Results

### 3.1 Longitudinal weights

**Figure 3:**
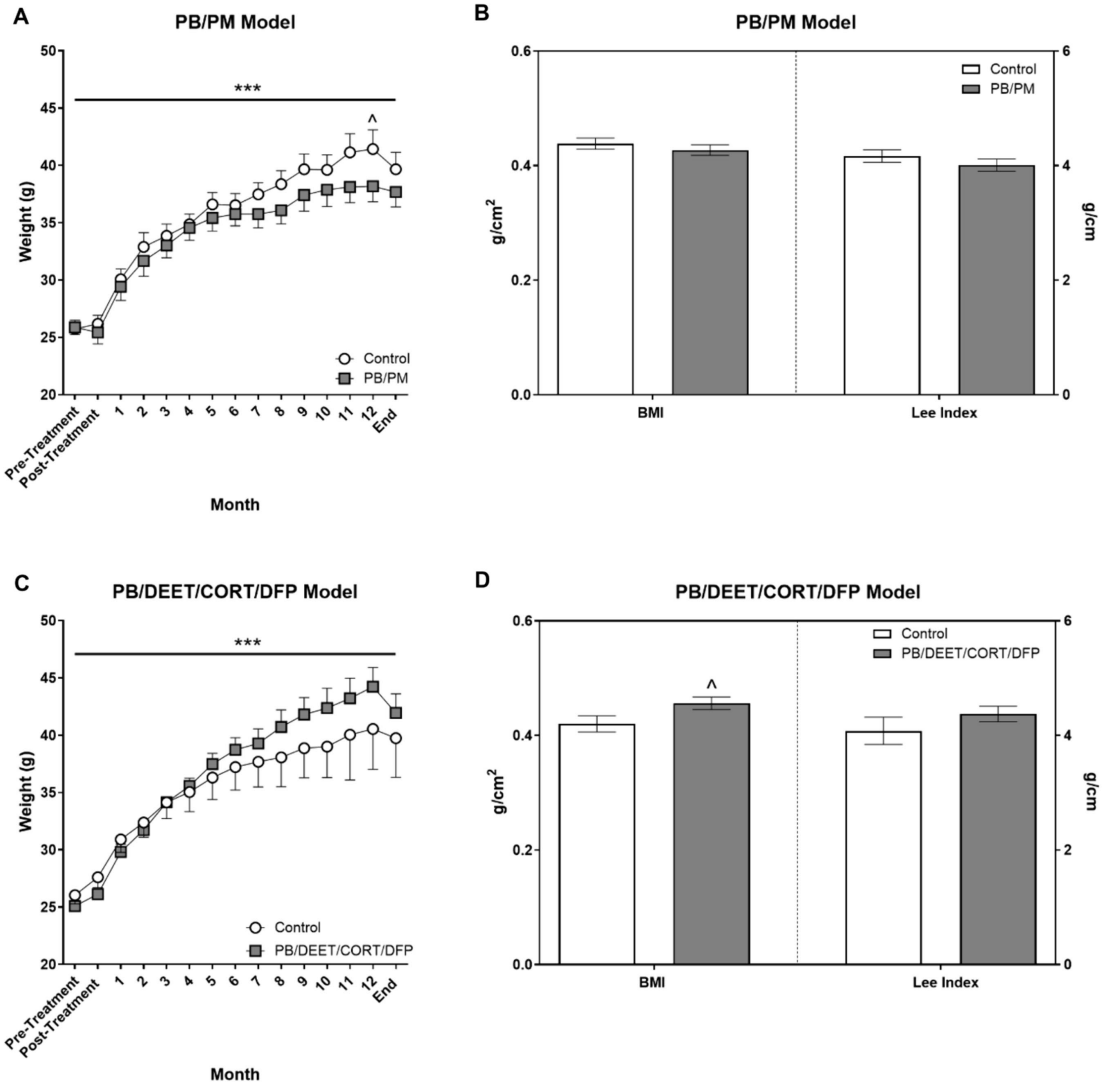
Monthly weights and calculated morphometrical parameters. **(A,C)** Weights were monitored and are presented for the start of the study (pre-treatment), directly after treatment administration cessation (PB/PM: 10 days; PB/DEET/CORT/DFP: 15 days), and monthly until the end of the study at 13.5 months. **(B,D)** Morphometrical parameters were evaluated by calculating the body mass index [BMI; Body weight (g)/length^2^ (cm^2^)] and the Lee index [body weight (g)/length (cm)]. Data are presented at ± SEM. Sample sizes were *n* = 6/group/timepoint for the PB/PM model and *n* = 5–6/group/timepoint for the PB/DEET/CORT/DFP model. ^***^Indicates a significant effect of time, *p* ≤ 0.001. ^^^Indicates a trending effect of treatment, *p* ≤ 0.10. CORT, corticosterone; DEET, N,N-diethyl-meta-toluamide; DFP, diisopropylfluorophosphate; g, grams; PB, pyridostigmine bromide; PM, permethrin.

Body weights were not different in either model at the beginning of the study (PB/PM model: control, 25.7 ± 0.76 vs. PB/PM, 25.9 ± 0.62, *p* ≥ 0.89; PB/DEET/CORT/DFP model: control 26.1 ± 0.77 vs. PB/DEET/CORT/DFP, 25.1 ± 0.70 *p* ≥ 0.38). In both models, weights significantly increased over time [PB/PM model: *F*(14, 140) = 162.24, *p* ≤ 0.001, Figure 3A; PB/DEET/CORT/DFP model: *F*(14, 137) = 76.20, *p* ≤ 0.001, Figure 3C]. In the PB/PM model, body weights were not significantly different between treatments throughout the study except for a numerical increase by the control group at 12 months (*p* = 0.10) (Figure 3A). An opposite numerical increase was observed in the PB/DEET/CORT/DFP treated mice (*p* = 0.13) (Figure 3C). Further, in the PB/PM model, there were no treatment differences for the calculated BMI or Lee indices (*p*’s ≥ 0.33) (Figure 3B). The Lee index, albeit numerically greater, was unaffected by treatment in the PB/DEET/CORT/DFP model (*p* ≥ 0.29). However, there was a strong trend for increased BMI in the PB/DEET/CORT/DFP treated mice [*t*(9) = 2.06, *p* = 0.07] (Figure 3D). At euthanasia, brain weights, both absolute and normalized to body weight, did not differ in either model (*p*’s ≥ 0.24, data not shown).

### 3.2 Prior PB/DEET/CORT/DFP exposure, but not PB/PM exposure, leads to overall brain volume reduction

**Figure 4:**
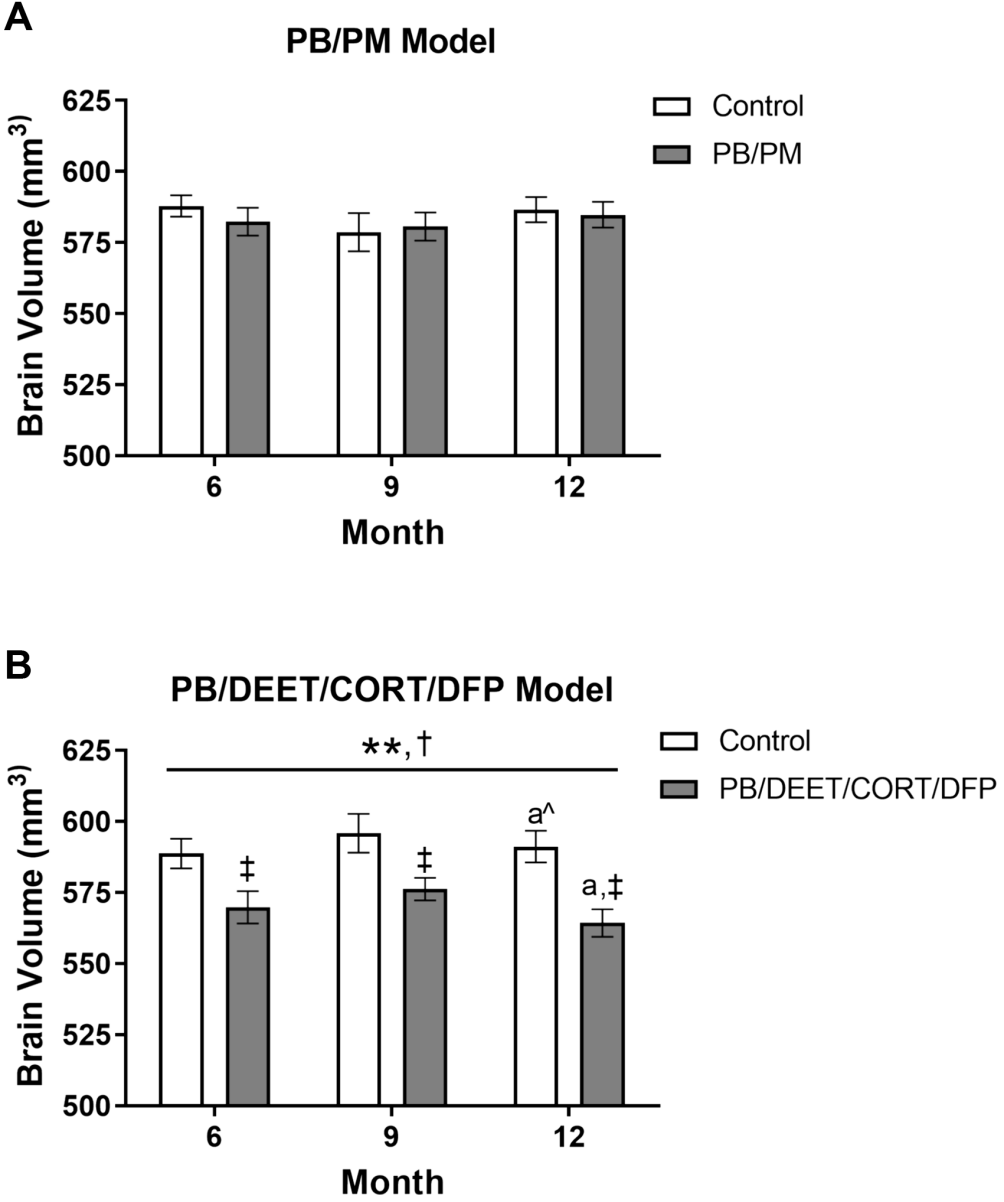
Longitudinal total brain volumes in two models of Gulf War Illness. Longitudinal measurement of total brain volume in **(A)** PB/PM and **(B)** PB/DEET/CORT/DFP models of GWI. Data are presented as mean ± SEM. Sample sizes were: PB/PM model, *n* = 6/group/timepoint; PB/DEET/CORT/DFP model, *n* = 5–6/group/timepoint. ^**^Indicates significant main effect of time where *p* ≤ 0.01. ^†^Indicates significant main effect of treatment, *p* ≤ 0.05, and ^‡^ denotes a significant pairwise comparison of treatment within a time point. ^a^ and ^a^^ denote a significant (*p* ≤ 0.05) and trending (*p* ≤ 0.10) pairwise comparison from the 9-month time point, respectively. CORT, corticosterone; DEET, N,N-diethyl-meta-toluamide; DFP, diisopropylfluorophosphate; PB, pyridostigmine bromide; PM, permethrin.

In the PB/PM model, there were no significant effects of time or treatment on overall brain volume (*p*’s ≥ 0.42) (Figure 4A). Over the course of the study, a slight 0.42% increase and 0.21% decrease in total brain volume were observed in the GWI and control groups, respectively (Supplementary Table S1). In the PB/DEET/CORT/DFP model, significant changes in global brain volume were observed for treatment [*F*(1, 19) = 8.55, *p* ≤ 0.05] (Figure 4B) and time [*F*(2, 19) = 7.22, *p* ≤ 0.01] (Figure 4B). Here, there were significant reductions in total brain volume for the PB/DEET/CORT/DFP group compared to control at the 6, 9, and 12-month time points (*p*’s ≤ 0.05), and this translated to an overall reduction of 0.08 and 0.96% in brain volume over the course of the study in the control and GWI groups, respectively (Supplementary Table S1). Within treatment, total brain volume was reduced significantly from 9 to 12 months in the PB/DEET/CORT/DFP (*p* ≤ 0.05) (Figure 4B) group; in the control mice, there was a trend for the age-related decrease of brain volume at 12 months (vs. 9 months, *p* = 0.07) (Figure 4B). This suggests GWI-related early loss of total brain volume, especially in the PB/DEET/CORT/DFP model, that is accelerated by age.

### 3.3 Ventricular enlargement is present in both models over time

**Figure 5:**
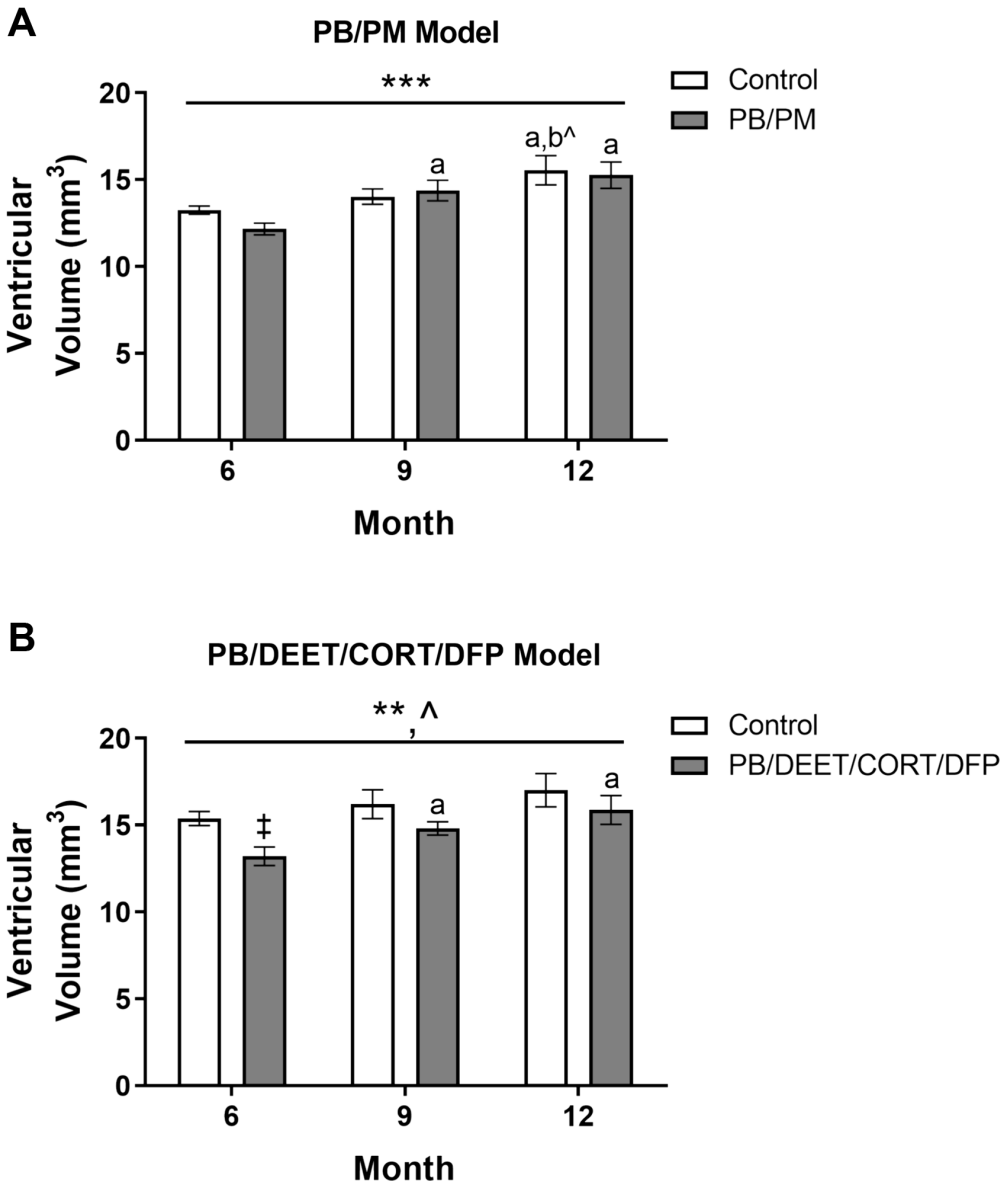
Longitudinal MRI analysis of ventricular volume in two models of Gulf War Illness. Longitudinal measurement of total ventricular volume (mm^3^) in **(A)** PB/PM and **(B)** PB/DEET/CORT/DFP models of GWI. Data are presented as mean ± SEM. Sample sizes were: PB/PM model, *n* = 6/group/timepoint; PB/DEET/CORT/DFP model, *n* = 5–6/group/timepoint. ^**^ and ^***^ indicate a significant main effect of time, *p* ≤ 0.01 and 0.001, respectively. ^^^Indicates a trending main effect of treatment, *p* = 0.08. ^a^ and ^b^ denote a significant pairwise comparison from the 6- and 9-month time points (*p* ≤ 0.05), respectively, whereas ^b^^ denotes a trending pairwise comparison from the 9-month time point (*p* = 0.08). ^‡^Denotes a significant pairwise comparison of treatment within a time point. CORT, corticosterone; DEET, N,N-diethyl-meta-toluamide; DFP, diisopropylfluorophosphate; PB, pyridostigmine bromide; PM, permethrin.

Total ventricular size (3rd, 4th, and lateral ventricles) was increased in the PB/PM [*F*(2, 30) = 10.87, *p* ≤ 0.001] (Figure 5A) model over time. An early increase was observed in the GWI group starting at 9 months (vs. 6 months, *p* ≤ 0.05), and ventricles became larger at 12 months (vs. 6 months, *p*’s ≤ 0.01). Additionally, an increase in ventricular size was observed in the control group at 12 months (vs. 6 months, *p* ≤ 0.05; vs. 9 months *p* = 0.08). By study end, this resulted in a 17 and 25% increase in volume from the 6-month timepoint in the control and GWI groups, respectively (Supplementary Table S1). Ventricular enlargement was also observed over time in the PB/DEET/CORT/DFP model [*F*(2, 19) = 7.41, *p* ≤ 0.01] (Figure 5B) in addition to a trending effect of treatment [*F*(1, 19) = 3.73, *p* = 0.08] (Figure 5B). Here, this treatment trend was driven by initially smaller ventricular volumes in the GWI group within the 6-month time point [*t*(10) = −3.25, *p* ≤ 0.01] (Figure 5B), an effect likely resulting from the decreased total brain volume in this model (Figure 4B). However, with age, significant increases in total ventricular size were seen in GWI mice at the 9-and 12-month time points (vs. 6, *p*’s ≤ 0.05). At the end of the study, the ventricular volume in the GWI group increased 20% from the 6-month time point, whereas the control increased 11% (Supplementary Table S1).

Individual ventricles were delineated to determine where these observed increases originated. In the PB/PM model, there was a significant volume increase in both the 4th ventricle [*F*(2, 30) = 4.25, *p* ≤ 0.05] and lateral ventricle [*F*(2, 30) = 12.93, *p* ≤ 0.001] over time, but not the 3rd ventricle (Table 1). Within the 4th ventricle, there was a significant volume increase in both groups at 9 months that was driven by the GWI group (vs. 6 months, *p* ≤ 0.05) (Table 1). Interestingly, this increase at 9 months was transient, as it did not persist to month 12 (*p* = 0.16) (Table 1). Lateral ventricle size gradually increased over time in both groups (*p*’s ≤ 0.05), and this was driven by early, significant increases in the GWI group at 9 months that persisted to 12 months (vs. 6 months, *p*’s ≤ 0.01) (Table 1) and a significant increase in the control group at 12 months (vs. 6 and 9 months, *p*’s ≤ 0.05) (Table 1).

**Table 1:** Longitudinal MRI assessment of ventricular and hindbrain area volumes in the PB/PM model of Gulf War Illness.

Region		Time point	Control (*n* = 6)	PB/PM (*n* = 6)
Ventricles	Lateral ventricle***	6	6.41 ± 0.22	5.50 ± 0.25
		9^a^	6.62 ± 0.40	7.24 ± 0.38^a^
		12^a,b^	8.13 ± 0.62^a,b^	8.16 ± 0.57^a^
	3rd ventricle	6	5.09 ± 0.18	5.00 ± 0.16
		9	5.38 ± 0.20	5.06 ± 0.25
		12	5.53 ± 0.14	5.20 ± 0.24
	4th ventricle*	6	1.75 ± 0.14	1.67 ± 0.10
		9^a^	2.03 ± 0.11	2.08 ± 0.06^a^
		12	1.89 ± 0.15	1.91 ± 0.13
Hindbrain	Cerebellum	6	75.85 ± 1.59	75.67 ± 1.31
		9	78.49 ± 1.24	77.17 ± 0.47
		12	76.08 ± 1.65	75.24 ± 2.26
	Medulla	6	32.04 ± 0.89	33.56 ± 0.66
		9	33.14 ± 0.37	32.36 ± 0.45
		12	33.36 ± 1.78	30.43 ± 1.63
	Pons^	6	22.64 ± 0.40	22.48 ± 0.51
		9	22.66 ± 0.50	21.96 ± 0.47
		12	21.37 ± 0.83	20.85 ± 1.05

This table depicts the longitudinal measurement (6, 9, and 12 months post-PB/PM treatment) of volume (mm^3^) in the ventricles and hindbrain. Sample sizes were *n* = 6/group/time point. Data are presented as mean ± SEM. ^*,**,^ and ^***^ indicate significant main effect of time where *p* ≤ 0.05, 0.01, and 0.001, respectively. ^a^ and ^b^ denotes significance from the 6-, and 9-month time point, respectively, *p* ≤ 0.05. ^^^Denotes a trending (*p* ≤ 0.06) effect of time. MRI, magnetic resonance imaging; PB, pyridostigmine bromide; PM, permethrin.

Within the PB/DEET/CORT/DFP model, the total ventricular enlargement was largely driven by significant treatment-related increases in the lateral ventricle over time {Treatment: [*F*(1, 19) = 7.62, *p* ≤ 0.05] and Time: [*F*(2, 19) = 15.33, *p* ≤ 0.001]; Table 2}. Lateral ventricle size gradually increased over time in both groups (control, 6 vs. 9 and 12 months, *p*’s ≤ 0.05), however, these increases were more pronounced within the GWI group (6 vs. 9 and 12 months, *p*’s ≤ 0.01 and 0.001, respectively). Interestingly, likely due to the decreased brain volume overall, lateral ventricle size within the GWI group was significantly smaller than the control group at all-time points (6 and 9 months, *p* ≤ 0.05, 12 months, *p* = 0.06). While there were numerical increases in the 3rd and 4th ventricles over time, none were significant (*p*’s ≥ 0.16).

**Table 2:** Longitudinal MRI assessment of the ventricular and hindbrain area volumes in the PB/DEET/CORT/DFP model of Gulf War Illness.

Region		Time point	Control (*n* = 5)	PB/DEET/CORT/DFP (*n* = 6)
Ventricles	Lateral ventricle***,^†^	6	8.02 ± 0.31	6.39 ± 0.28^‡^
		9^a^	8.98 ± 0.54^a^	7.63 ± 0.39^‡,a^
		12^a^	9.60 ± 0.66^a^	8.01 ± 0.40^^,a^
	3rd ventricle	6	5.49 ± 0.12	4.86 ± 0.26
		9	5.46 ± 0.30	5.27 ± 0.16
		12	5.38 ± 0.43	5.74 ± 0.52
	4th ventricle	6	1.88 ± 0.11	1.96 ± 0.11
		9	1.77 ± 0.16	1.91 ± 0.16
		12	2.04 ± 0.14	2.13 ± 0.12
Hindbrain	Cerebellum*,^†^	6	78.49 ± 1.73	74.80 ± 0.76^‡^
		9	77.07 ± 1.15	74.26 ± 0.93^^^
		12^a,b^	76.19 ± 0.95^a^	72.51 ± 0.74
	Medulla^†^	6	33.78 ± 0.50	31.77 ± 0.85^‡^
		9	33.56 ± 0.73	31.53 ± 0.59^‡^
		12	33.94 ± 0.34	31.80 ± 0.69^^^
	Pons	6	23.13 ± 0.29	21.85 ± 0.60^^^
		9	23.13 ± 0.29	21.61 ± 0.67^^^
		12	22.64 ± 0.50	22.48 ± 0.52

This table depicts the longitudinal measurement (6, 9, and 12 months post-PB/DEET/CORT/DFP treatment) of volume (mm^3^) in the ventricles and hindbrain. Sample sizes were *n* = 5–6/group/time point. Data are presented as mean ± SEM. ^***^ and ^*^ indicate a significant main effect of time where *p* ≤ 0.001 or 0.05, respectively. ^†^Denotes a significant (*p* ≤ 0.05) main effect of treatment. ^‡^ and ^^^ denote a significant or trending treatment effect within a time point where *p* ≤ 0.05 or 0.09, respectively. ^a^ and ^b^ denote significance from the 6- and 9-month time point, respectively, *p* ≤ 0.05. CORT, corticosterone; DEET, N,N-diethyl-meta-toluamide; DFP, diisopropylfluorophosphate; MRI, magnetic resonance imaging; PB, pyridostigmine bromide.

### 3.4 Cortical volume and thickness are decreased in both models over time

**Figure 6:**
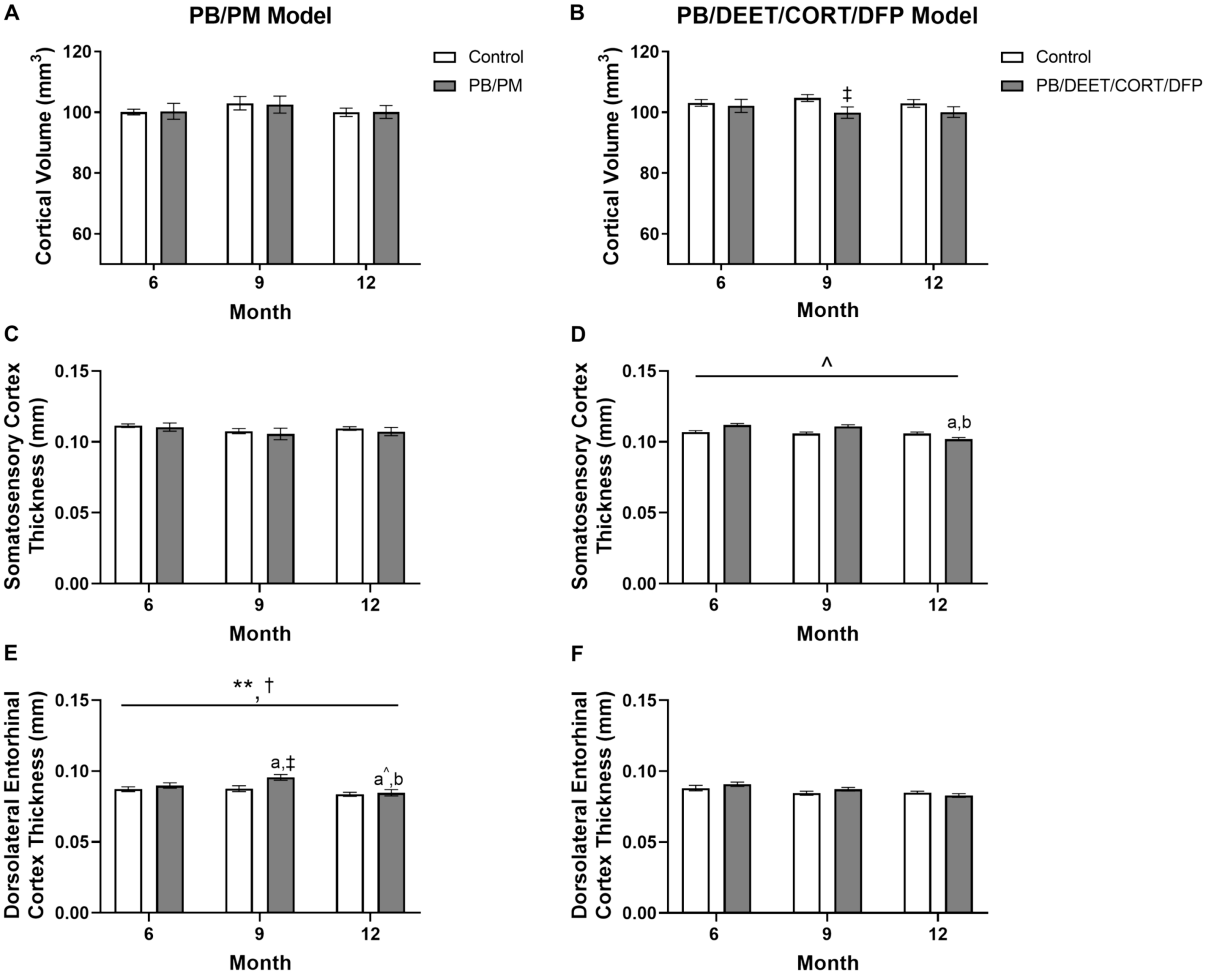
Longitudinal MRI analysis of cortical volume and thickness in two models of Gulf War Illness. Longitudinal measurement of cortical volumes (mm^3^) in **(A)** PB/PM and **(B)** PB/DEET/CORT/DFP models of GWI. Longitudinal measurement of thickness (in mm) of the somatosensory and dorsolateral entorhinal cortices were conducted in **(C,E)** PB/PM and **(D,F)** PB/DEET/CORT/DFP models of GWI, respectively, over the course of 12 months. Data are presented as mean ± SEM. Sample sizes were: PB/PM model, *n* = 6/group/timepoint; PB/DEET/CORT/DFP model, *n* = 5–6/group/timepoint. ^**^Indicates a significant main effect of time where *p* ≤ 0.01. ^†^Indicates significant main effect of treatment, *p* ≤ 0.05. ^‡^Denotes a significant pairwise comparison of treatment within a time point. ^a^ and ^b^ denote a significant pairwise comparison from the 6- and 9-month time points, respectively, *p* ≤ 0.05. ^a^^Denotes a trending effect from the 6-month time point, *p* ≤ 0.10. CORT, corticosterone; DEET, N,N-diethyl-meta-toluamide; DFP, diisopropylfluorophosphate; PB, pyridostigmine bromide; PM, permethrin.

There were no significant time or GWI treatment effects for cortical volume observed in the PB/PM model (*p*’s ≥ 0.36) (Figure 6A); over time, cortical volume decreased by 0.09 and 0.18% in the control and GWI groups, respectively (Supplementary Table S1). Similarly, in the PB/DEET/CORT/DFP model, the age effect on cortical volume was not significant (*p*’s ≥ 0.64). The percent change from 6 to 12 months was 0.56 and 2% for the control and GWI groups, respectively (Supplementary Table S1). Notably, a significant treatment effect was observed at 9 months where there was a reduction in cortical volume in the GWI mice compared to control [*t*(10) = −2.20, *p* ≤ 0.05] (Figure 6B); a similar, non-significant trend was observed at 12 months (Figure 6B).

To complement the volume analyses, cortical thickness measurements were conducted across the frontal, motor, auditory, somatosensory, and dorsolateral entorhinal cortices in both models. In the PB/PM model, there were no significant effects of treatment observed in the somatosensory cortex (Figure 6C). In the frontal cortex, there was a transient decrease in thickness within the GWI group at 9 months (vs. 6 months, [*t*(10) = 2.68, *p* ≤ 0.05]; Table 3) that did not persist to 12 months. A trending decrease in motor cortex thickness within the control group at 12 months {vs. 6 months, [*t*(10) = 2.08, *p* = 0.06; Table 3]} also resulted in a treatment trend when controls were compared with the GWI group [*t*(10) = −2.00, *p* = 0.07] (Table 3). Similar reductions were apparent within the auditory cortex over time [*F*(2, 30) = 4.62, *p* ≤ 0.05]; these effects were driven largely by numerical decreases in both groups at month 12 (*vs* 6 months, *p*’s ≤ 0.10) (Table 3). Significant thinning of the dorsolateral entorhinal cortex was apparent with age [*F*(2, 30) = 7.78, *p* ≤ 0.01] (Figure 6E) and treatment [*F*(2, 30) = 6.11, *p* ≤ 0.05] (Figure 6E). Interestingly, there was an increase in thickness at 9 months in the GWI group (vs. 6 months, *p* ≤ 0.05) that was significantly higher than the control group (*p* ≤ 0.01). This increase was transient, as thickness decreased by 12 months (vs. 9 months, *p* ≤ 0.01 and vs. 6 months, *p* = 0.07; Figure 6E) and was similar to the control group.

**Table 3:** Longitudinal analysis of cortical thickness in two models of Gulf War Illness.

		PB/PM model		PB/DEET/CORT/DFP model	
Region	Time point	Control (*n* = 6)	GWI (*n* = 6)	Control (*n* = 5–6)	GWI (*n* = 6)
Frontal cortex	6	0.30 ± 0.006	0.30 ± 0.006	0.29 ± 0.006	0.29 ± 0.010
	9	0.29 ± 0.009	0.28 ± 0.003^a^	0.28 ± 0.007	0.31 ± 0.005
	12	0.29 ± 0.009	0.30 ± 0.007^b^^	0.29 ± 0.010	0.29 ± 0.010
Motor cortex	6	0.12 ± 0.001	0.11 ± 0.002	0.12 ± 0.002	0.12 ± 0.002
	9	0.12 ± 0.003	0.11 ± 0.002	0.12 ± 0.003	0.12 ± 0.001
	12	0.11 ± 0.002^a^^	0.12 ± 0.001^^^	0.13 ± 0.000	0.12 ± 0.002
Auditory cortex	6	0.10 ± 0.005	0.10 ± 0.003	0.09 ± 0.001	0.08 ± 0.002
	9	0.09 ± 0.004	0.10 ± 0.002	0.09 ± 0.003	0.08 ± 0.002
	12	0.09 ± 0.003^a^^	0.09 ± 0.002^a^^	0.09 ± 0.003	0.08 ± 0.002

Cortical thickness measurements (in mm) were conducted across the frontal, motor, auditory, somatosensory, and dorsolateral entorhinal in the PB/PM and PB/DEET/CORT/DFP models of GWI over the course of 12 months. Data are presented as mean ± SEM. Sample sizes were: PB/PM model, *n* = 6/group/timepoint; PB/DEET/CORT/DFP model, *n* = 5–6/group/timepoint. ^a^ and ^b^ denote a significant pairwise comparison from the 6- and 9-month time points, respectively, *p* ≤ 0.05. ^^^ denotes a trending effect of treatment, whereas ^a^^ and ^b^^ indicate a trending effect from 6 and 9 months, respectively, *p* ≤ 0.10. CORT, corticosterone; DEET, N,N-diethyl-meta-toluamide; DFP, diisopropylfluorophosphate; PB, pyridostigmine bromide; PM, permethrin.

In the PB/DEET/CORT/DFP model, there were no significant changes in cortical thickness observed with treatment or over time in the frontal, motor, auditory, or dorsolateral entorhinal cortices (Table 3; Figure 6F). However, there was a trending decrease in thickness over the course of 12 months within the somatosensory cortex [*F*(2, 19) = 2.95, *p* = 0.08] (Figure 6D); this numerical decrease was driven by significant decreases in thickness within the GWI group for this region at 12 months {vs. 6 months: [*t*(10) = −2.96, *p* ≤ 0.05] and vs. 9 months: [t(10) = −2.48, *p* ≤ 0.05]; Figure 6D).

### 3.5 Reductions in hippocampal volumes are present in both models over time

**Figure 7:**
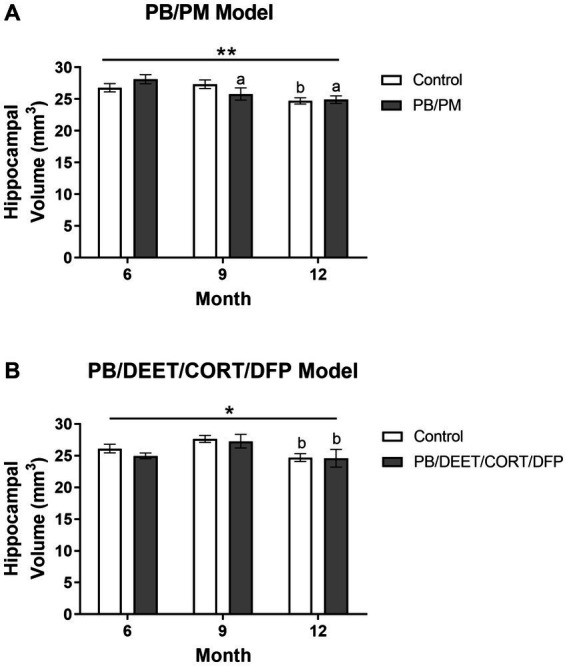
Longitudinal MRI analysis of hippocampal volume in two models of Gulf War Illness. Longitudinal measurement of hippocampal volumes in **(A)** PB/PM and **(B)** PB/DEET/CORT/DFP models of GWI. Data are presented as mean ± SEM. Sample sizes were: PB/PM model, *n* = 6/group/timepoint; PB/DEET/CORT/DFP model, *n* = 5–6/group/timepoint. ^*^ and ^**^ indicate a significant main effect of time where *p* ≤ 0.05 and 0.01, respectively. ^a^ and ^b^ denote a significant pairwise comparison from the 6- and 9-month time point, respectively, *p* ≤ 0.05. CORT, corticosterone; DEET, N,N-diethyl-meta-toluamide; DFP, diisopropylfluorophosphate; PB, pyridostigmine bromide; PM, permethrin.

Reduced hippocampal volume was present over time in the PB/PM [*F*(2, 30) = 7.39, *p* ≤ 0.01] (Figure 7A) and PB/DEET/CORT/DFP [*F*(2, 19) = 5.24, *p* ≤ 0.05] (Figure 7B) models. Pairwise comparisons revealed that hippocampal volume in the PB/PM model began to decline in the PB/PM group from month 6 to 9 (*p* ≤ 0.05) (Figure 7A) and continued to month 12 (*p* ≤ 0.01) (Figure 7A). In the control group, this effect was only observed at 12 months (9 vs. 12 months, *p* ≤ 0.05) (Figure 7A), highlighting an earlier onset of the decrease driven by GWI treatment. In the PB/DEET/CORT/DFP model, both groups had reduced hippocampal volumes at 12 months (9 vs. 12 months, *p*’s ≤ 0.05) (Figure 7B). Within the PB/PM model, the overall percent change in volume from 6 to 12 months was 7.75 and 11.40% in the control and GWI groups, respectively (Supplementary Table S1). Within the PB/DEET/CORT/DFP model, the percentage change in volume from 6 to 12 months was 5.03 and 2.03% in the control and GWI groups, respectively (Supplementary Table S1).

### 3.6 Alterations in the hindbrain over time in both models

**Figure 8:**
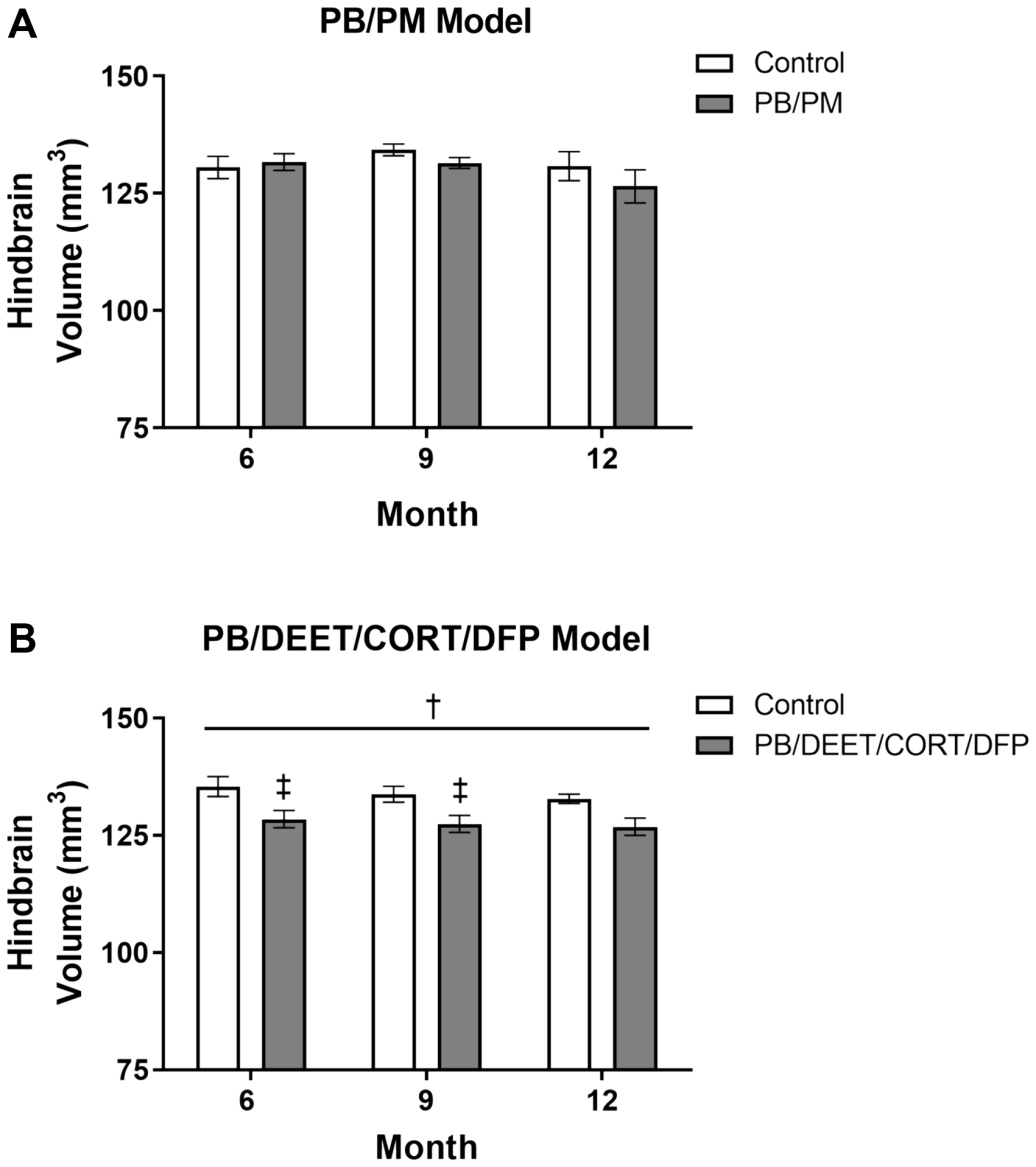
Longitudinal MRI analysis of the hindbrain volume in two models of Gulf War Illness. Longitudinal measurement of the hindbrain (cerebellum, pons, and medulla) volumes in the **(A)** PB/PM and **(B)** PB/DEET/CORT/DFP models of GWI. Data are presented as mean ± SEM. Sample sizes were *n* = 6/group/timepoint for the PB/PM model and *n* = 5–6/group/timepoint for the PB/DEET/CORT/DFP model. ^†^Denotes a significant main effect of treatment (*p* ≤ 0.05), and ^‡^ indicates a significant pairwise comparison of treatment within a time point (*p* ≤ 0.05). CORT, corticosterone; DEET, N,N-diethyl-meta-toluamide; DFP, diisopropylfluorophosphate; PB, pyridostigmine bromide; PM, permethrin.

Overall, no significant effects for time or treatment were observed in the hindbrain (cerebellum, medulla, and pons) in the PB/PM model (*p*’s ≥ 0.22; Figure 8A). However, the percentage change in hindbrain from 6 to 12 months indicated an overall reduction of 3.95% in the GWI group and an increase of 0.21% in the control group. A numerical (*p* = 0.12) reduction in hindbrain volume was observed over time within the PB/DEET/CORT/DFP model; this translated to an overall reduction of 2.15 and 1.27% in the control and GWI groups, respectively from 6 to 12 months. Notably, the hindbrain volume of the GWI group was significantly smaller than the control group [*F*(1, 19) = 8.46, *p* ≤ 0.05] (Figure 8B), particularly within 6- and 9-month time points (*p*’s ≤ 0.05) (Figure 8B), which might partly explain the total brain volume difference between groups in this model.

Similar to the ventricular volumetric analysis, the individual areas of the hindbrain (cerebellum, medulla, and pons) were analyzed to gather more specific information on these brain regions over time. Interestingly, cerebellar volumes fluctuated slightly in the PB/PM model over the course of 12 months (Table 1) and were significantly decreased over time in the PB/DEET/CORT/DFP model [*F*(2, 19) = 5.37, *p* ≤ 0.05] (Table 2). A significant reduction in cerebellar volume was observed at 12 months within the control group of the PB/DEET/CORT/DFP model (vs. 6 months, *p* ≤ 0.05); no significant reductions were observed in the GWI group with age (Table 2). Notably, a significant main effect of treatment was present within the cerebellum [*F*(1, 19) = 6.30, *p* ≤ 0.05] where cerebellar volume was smaller within the GWI group compared to control, particularly at months 6 and to a lesser extent, 9 (*p*’s ≤ 0.05 and 0.08, respectively).

Within the brainstem, both medullary and pontine volumes numerically decreased over the course of 12 months in the PB/PM model (Table 1). In the pons, there was a trending effect for reduction in volume over time [*F*(2, 30) = 2.70, *p* = 0.08]. However, this reduction was not significant in the medulla (*p* = 0.66). Within the PB/DEET/DFP/CORT model, there were significant and trending main effects of treatment for the medulla [*F*(1, 19) = 8.02, *p* ≤ 0.05] and pons [*F*(1, 19) = 3.40, *p* = 0.09], respectively (Table 2). Here, medullary volume was significantly smaller in the GWI group than controls at the 6-and 9-month time points (*p* ≤ 0.05) and trended at the 12-month time point (*p* = 0.07). Similar strong trends were observed in the pons where volume was smaller in the GWI group compared to control at 6 and 9 months (*p*’s = 0.08 and 0.06, respectively).

### 3.7 Behavior analyses

#### 3.7.1 Open field test

**Figure 9:**
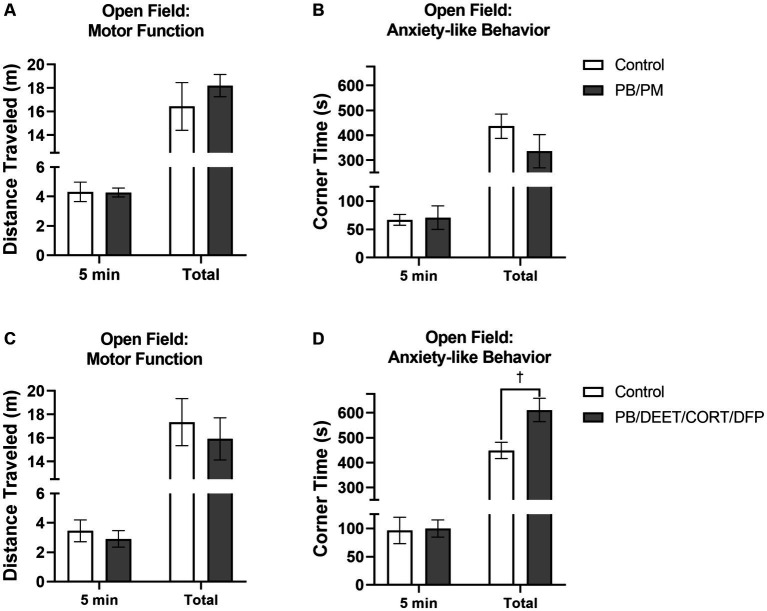
Open field test. Motor and mood effects were evaluated using the open field test 13 months post GWI chemicals exposure. Motor function was examined by the distance traveled **(A,C)** for the first 5 min and total 30 min. Anxiety-like behavior was evaluated by evaluating the **(B,D)** time spent in the corners of the arena. Data are presented as mean ± SEM. Sample sizes were *n* = 6/group/timepoint for the PB/PM model and *n* = 5–6/group/timepoint for the PB/DEET/CORT/DFP model. ^†^Indicates a significant treatment difference, *p* ≤ 0.05.

The open field test was used to gather insights into motor function and anxiety-like behavior in these mice at 13 months post study initiation. As expected, all mice habituated to the OF arena as evident by decreases in distance traveled over time [PB/PM model, *F*(5, 50) = 9.09, *p* ≤ 0.001; data not shown] and PB/DEET/CORT/DFP model, [*F*(5,50) = 9.90, *p* ≤ 0.001, data not shown]. In the PB/PM model, both groups traveled similar distances during the first 5 and total 30 minutes; at 30 min, the slightly greater distance covered by the GWI mice was not significant (*p* = 0.44) (Figure 9A). In the PB/DEET/CORT/DFP model, the numerically shorter distance that the GWI mice traveled during the first 5 min and the total 30 min was also not significant (*p*’s ≥ 0.56) (Figure 9C).

Anxiety-like behavior was measured by examining the time spent in the corners of the OF arena. There were no significant impacts on anxiety-like behavior within the first 5 min of the test for either model (*p*’s ≥ 0.87; Figures 9B,D). Interestingly at 30 min, prior GWI exposure led to more anxiety-like behavior compared to controls in the PB/DEET/CORT/DFP model as these mice spent more time in the corners [*t*(9) = 2.72, *p* ≤ 0.05] (Figure 9D); this was not the case in the PB/PM model (*p* = 0.25) (Figure 9B).

#### 3.7.2 Temperature response stress test

**Figure 10:**
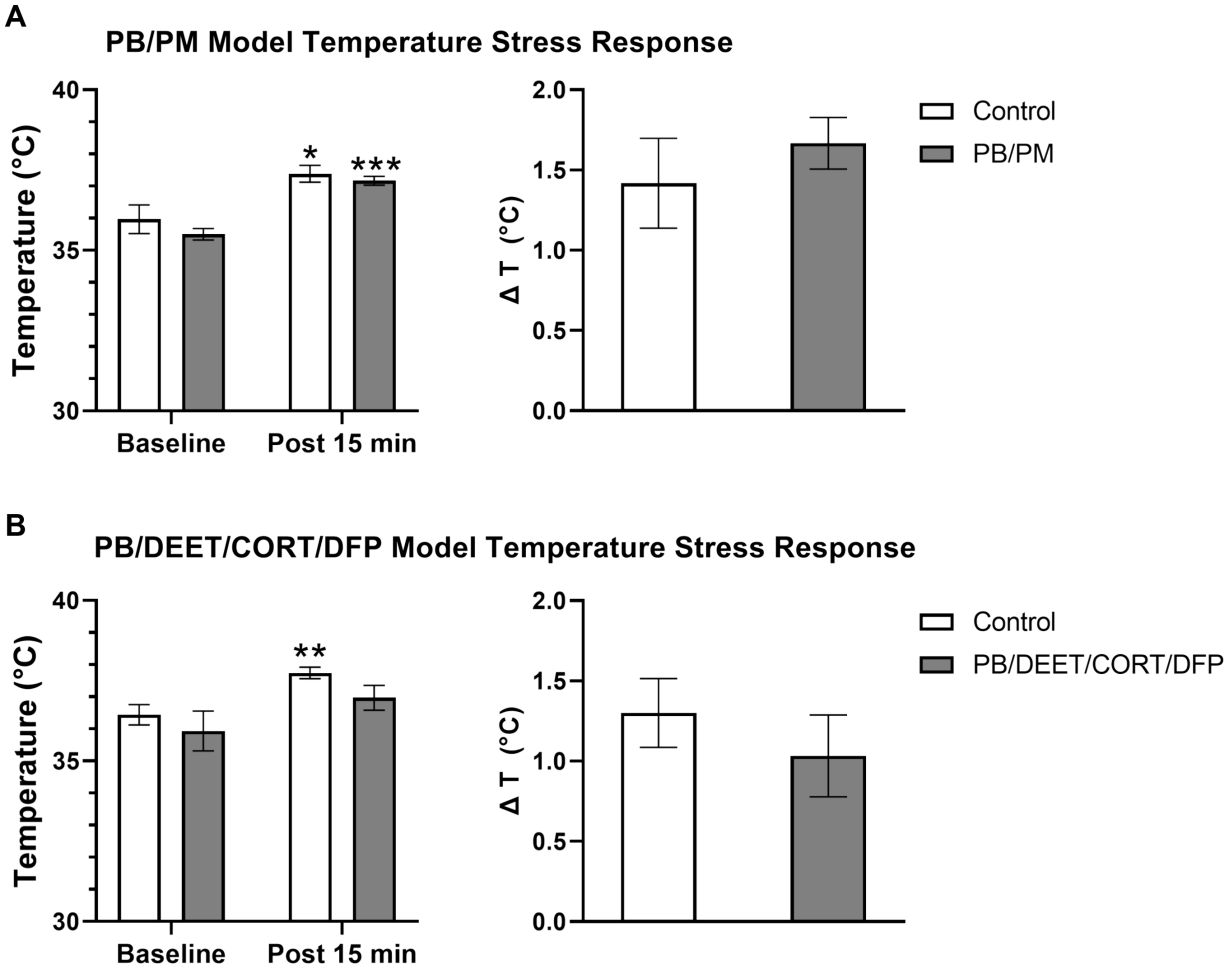
Temperature response stress test conducted 13 months post GWI chemicals exposure. The temperature response stress test was used to assess stress-induced hyperthermia following two temperature measurements (baseline and post 15 min). Data are presented as mean ± SEM. Sample sizes were: PB/PM model, *n* = 6/group/timepoint; PB/DEET/CORT/DFP model, *n* = 5–6/group/timepoint. ^*,**,^ and ^***^ indicate a significant effect of time where *p* ≤ 0.05, 0.01, and 0.001, respectively. CORT, corticosterone; DEET, N,N-diethyl-meta-toluamide; DFP, diisopropylfluorophosphate; PB, pyridostigmine bromide; PM, permethrin; T, temperature.

All mice in both models exhibited increases in body temperature indicative of stress-induced (probe insertion) hyperthermia. Within the PB/PM model, there were no significant treatment differences between groups, just significant increases in temperature from the baseline reading for both groups {control [*t*(10) = −2.73, *p* ≤ 0.05]; PB/PM [*t*(10) = −7.28, *p* ≤ 0.001]; Figure 10A}. Body temperature increased by 1.42 ± 0.28°C and 1.67 ± 0.16°C, respectively, for the control and GWI mice; no significant treatment difference was observed for the change in temperature as well (*p* = 0.46) (Figure 10A).

Similarly, within the PB/DEET/CORT/DFP model, there were no significant treatment differences among groups. Although not significant, at baseline, the PB/DEET/CORT/DFP group had a numerically lower temperature compared to the control group. The control mice exhibited a significant increase in temperature [*t*(8) = −3.58, *p* ≤ 0.01], but the GWI group, despite having a lower baseline temperature, had only a numerical elevation in body temperature (*p* = 0.19) (Figure 10B). Body temperature increased by 1.30 ± 0.21°C and 1.03 ± 0.26°C, respectively, for the control and GWI mice; no significant difference was observed for the change in temperature between treatment groups (*p* = 0.46) (Figure 10B).

## 4 Discussion

**Figure 11:**
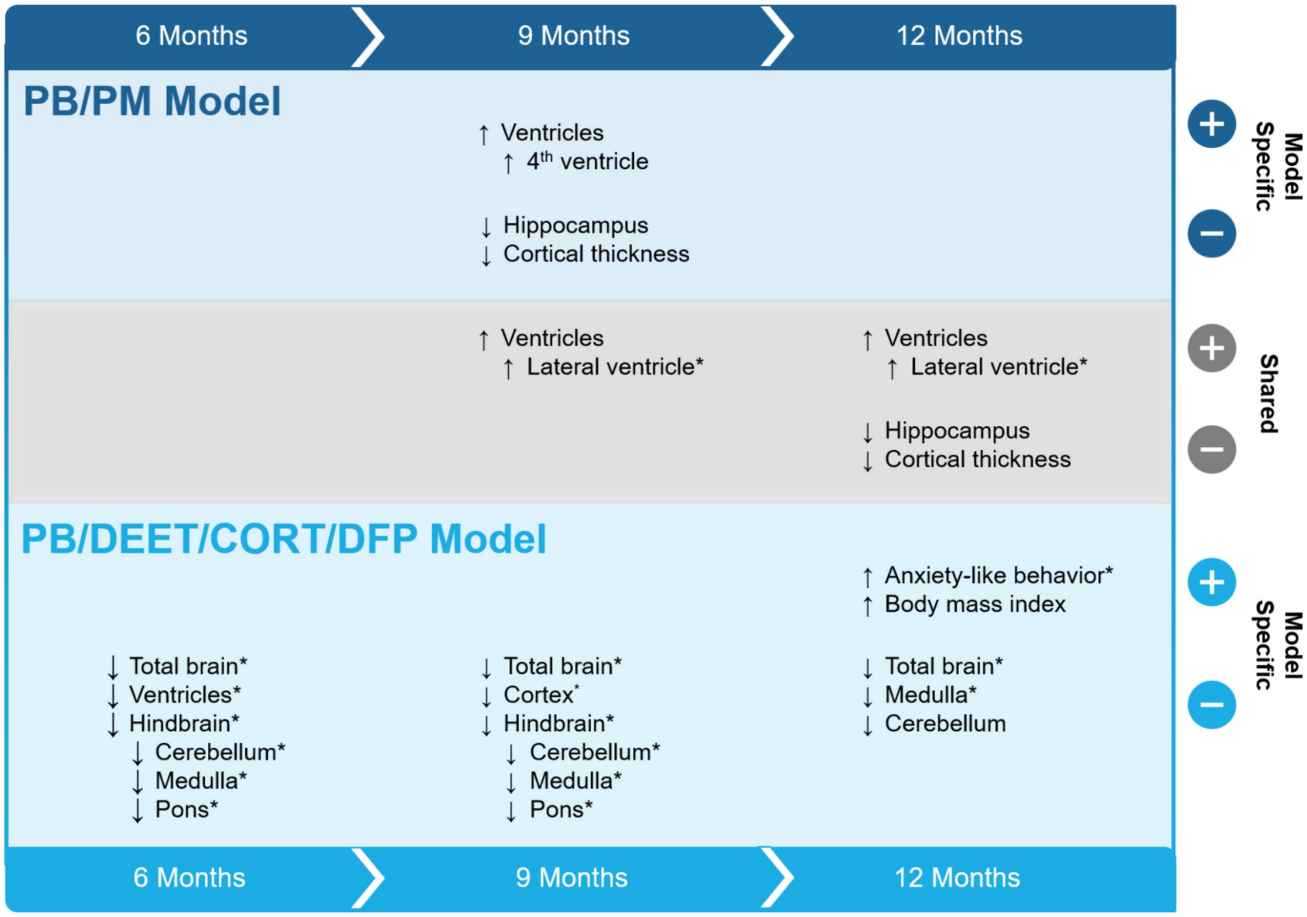
Summary of study results. The effects of two Gulf War Illness models on brain volume over the course of 12 months are shown. Model-specific and shared effects are presented as either increases or decreases in volume at the 6-, 9-, and 12-month time points. ^*^Indicates a treatment difference within the GWI model and is plotted in the direction of the GWI effect. CORT, corticosterone; DEET, N,N-diethyl-meta-toluamide; DFP, diisopropylfluorophosphate; PB, pyridostigmine bromide; PM, permethrin; T, temperature.

This study sought to evaluate the progression of structural brain alterations in two GWI preclinical models over the course of 12 months. The results, summarized in Figure 11, highlight similarities and distinctions between these exposure paradigms and aid in the understanding of GWI pathogenesis and endophenotypes. Major similarities among the models include ventricular enlargement and reductions in global brain and hippocampal volumes with age. Key differences were model specific, in which the PB/DEET/CORT/DFP model led to reduced brainstem volumes and an early and persistent loss of total brain volume, while the PB/PM model produced reductions in cortical volume and thickness with age. Behaviorally, at 13 months, motor function was largely preserved in both models. However, the GWI mice in the PB/DEET/CORT/DFP model exhibited an elevation in anxiety-like behavior.

Weights were measured for overall health monitoring throughout this study. There were no significant treatment differences over the course of the study (13.5 months). However, PB/PM treated mice weighed numerically less than controls, while PB/DEET/CORT/DFP mice gained more weight over time and trended higher in BMI than controls at the end of the study. These results align with the higher rates of overweight and obesity observed in GWI veterans (Coughlin et al., 2011; Breland et al., 2017), and suggest that prior exposure to this model’s specific chemicals (PB/DEET/DFP) in addition to stress (CORT in this study) may impact weight (e.g., metabolic function) later in life. In fact, it was observed with the temperature response stress test that the GWI mice in the PB/DEET/CORT/DFP model had lower baseline body temperatures and had the smallest elevation in temperature compared to the other groups. This suggests that these mice may have an altered metabolism due to weight gain or potential structural changes within the hypothalamus, including blunted HPA axis (Carmo-Silva and Cavadas, 2017). While this study was MRI-focused and did not dive into metabolic mechanisms or evaluate the hypothalamus, studies show that higher BMIs and obesity are associated with brain structural changes (e.g., reduced volumes; Ward et al., 2005), neuroinflammation, changes in cognitive function, and increased risk of neurological diseases, such as dementias or Alzheimer’s Disease (Whitmer et al., 2008; O’Brien et al., 2017). Further investigations into fat distribution and metabolic dysregulation in a model-specific context are warranted with increased sample sizes appropriate for such investigations.

Many of the effects observed in this study occurred over time, indicating that normal aging is an important factor to consider. In fact, numerous clinical studies have shown that with age, particularly beginning around middle age, global and regional brain volumes decrease in size (Gur et al., 1991; Murphy et al., 1992; Scahill et al., 2003). At the time of study completion (13.5 months post exposures), mice were approximately 15 months old, an age that corresponds to late middle-age in humans and the current status of many GWI veterans. Of note, at the end of the study, there were no differences in either model for gross wet brain weight. However, MRI analysis did reveal longitudinal alterations in global brain volume over time that were most pronounced at the latest scanning (12 months), particularly within the PB/DEET/CORT/DFP model. It has been shown that with age and age-related diseases, such as Alzheimer’s and Parkinson’s, increases in brain water are associated with gray matter atrophy and the breakdown of white matter (Maier-Hein et al., 2015; Ofori et al., 2015; Chad et al., 2018; Gullett et al., 2020). Thus, the lack of changes in brain weight at the end of this study might be an indicator of increased extracellular water compensating for parenchymal decreases. Indeed, significant treatment differences were observed for overall brain volume in the PB/DEET/CORT/DFP model, in which GWI mice exhibited smaller brain volumes compared to control at each time point examined; however, reductions in brain volume within controls did not appear until the 12-month time point. In the PB/PM model, the enlargement of ventricles over time may explain the lack of any discernible change in total brain volume; in fact, within the GWI group, there was a 0.42% increase in total brain volume that may stem from the 25% increase in ventricular size at 12 months. Further, compared to the PB/DEET/CORT/DFP model, the hippocampus was the only brain area examined that significantly decreased over time in the PB/PM model. This suggests the combination of chemicals utilized in this model do not globally impact brain structures, at least at the time points examined. In contrast, in the PB/DEET/CORT/DFP model, the effects observed over the course of the study were largely driven by volume reductions in the GWI group, indicating a treatment dependent effect that may also be compounded by normal aging. Investigations at later stages of life in these models may shed more light on the impact of aging on brain structure in the context of GWI.

It has been shown that ventricular size increases with age (Scahill et al., 2003; Preul et al., 2006) and is associated with declines in brain structure (e.g., cortical thinning) and consequently, neurobehavioral function like cognitive performance (Preul et al., 2006; Carmichael et al., 2007a, 2007b). Here, total ventricular volume was increased at 12 months in both groups of the PB/PM model, albeit to a less degree in the control group (17% control vs. 25% GWI). Similarly, ventricular volume enlarged with age in the PB/DEET/CORT/DFP model. While GWI mice in this model initially had significantly smaller ventricular volume compared to control mice, a pronounced enlargement with age was observed as early as month 9 that persisted to month 12. In fact, ventricular size increased by 20% in the GWI group, almost twice more than the controls. To further characterize where these increases in ventricular volume occurred, ventricles were delineated by the lateral, 3rd, and 4th ventricles and examined. Notably, these measures revealed similarities and distinctions across the evaluated models. Neither model produced significant effects of the 3^rd^ ventricle. However, lateral ventricle size significantly increased with age in both models starting at 9 months post exposures, and was more pronounced in both GWI groups. The changes observed PB/PM model at 12 months align with another GWI study investigating volumetric changes within this region at 10 months (Wu et al., 2021). Additionally, there was an increase in 4th ventricle size within the PB/PM model at the 9-month time point for GWI mice, which was not present at 12 months. This, coupled with the enlargement of the lateral ventricle, suggests a potential effect of neuroinflammation within this region and model. Studies have linked inflammation with transient overproduction of cerebral spinal fluid (CSF) and ventricular enlargement (Lepore et al., 2013; Millward et al., 2020). An earlier study utilizing the PB/PM model found GWI-specific increases in neuroinflammation at this time point as well as behavioral alterations (Carpenter et al., 2021). It is notable that areas containing CSF may contribute to structural changes in gray/white matter (Resnick et al., 2000; Scahill et al., 2003; Preul et al., 2006).

Regional alterations in gray matter were also apparent within these models over time. The PB/DEET/CORT/DFP model produced slight decreases in cortical volume and marginal cortical thinning was observed in both models with age. In the PB/PM model, the GWI increase in ventricular volume and age-associated cortical thinning, particularly in the frontal and dorsolateral entorhinal cortices, could explain the cognitive aberrations seen previously in this model (Zakirova et al., 2015; Zakirova et al., 2016; Carpenter et al., 2021) and GWI veterans. There was a transient decrease in thickness for the frontal cortex at 9 months in the GWI group, which aligns to behavioral aberrations in cognitive performance seen previously in this model at this time point (Carpenter et al., 2021). Interestingly, this effect did not persist beyond 9 months, and warrants further evaluation. In the PB/DEET/CORT/DFP model, thinning of the somatosensory cortex was observed with age and was more prominent in the GWI treated group. This aligns with behavioral outcomes from our earlier studies that identified significant impairments in the sticker removal test (Carpenter et al., 2021; Carpenter et al., 2022). The sticker removal test assesses sensorimotor function, which is negatively affected in veterans with GWI (Axelrod and Milner, 1997; Proctor et al., 2006; Toomey et al., 2009). The present results strengthen the connection between GWI chemicals exposure, cortical impact, and behavior.

Both models saw reductions in hippocampal volumes over time and in all groups, suggestive of an age-related volume reduction. Among models, there was a greater reduction in hippocampal volume for the GWI group in the PB/PM model (11%) than the PB/DEET/CORT/DFP model (2%) at 12 months. In the PB/DEET/CORT/DFP model, both GWI and control groups decreased at similar rates at 12 months. However, in the PB/PM model this reduction was observed at 9 months only in the GWI treated group, indicating that this prior PB/PM exposure impacts this region earlier. These data are in line with findings from GWI studies depicting volume reductions in rats (Wu et al., 2021) and in the GWI veteran population (Chao et al., 2010). Additionally, the significant thinning of the dorsolateral entorhinal cortex over time in this model, particularly within the GWI group, may further compound hippocampal effects, such as cognitive, memory, and synaptic transmission deficits that have been observed in a previous studies utilizing this paradigm (Brown et al., 2021b; Carpenter et al., 2021). Hindbrain atrophy has also been observed in GWI veterans (Christova et al., 2017) and may contribute to the various symptoms experienced by these individuals. Atrophy in the areas of the hindbrain could potentially impact vital functions such as motor control, sleep patterns, and respiration (Moini and Piran, 2020). Here, there were numerical reductions in the hindbrain with age in both models, but effects were more pronounced in the PB/DEET/CORT/DFP model. In the PB/DEET/CORT/DFP model there was a significant treatment difference in volume compared to control at all-time points examined. The hindbrain was delineated into its key regions (e.g., cerebellum, pons, and medulla) to identify any structural changes that may be in line with previously observed behavioral changes in these models and clinical symptomology of veterans with GWI. Interestingly, cerebellar volumes fluctuated within the PB/PM model, and ultimately were numerically reduced at 12 months; a similar reduction was observed in the PB/DEET/CORT/DFP model and these effects are attributed to aging. Further within the PB/DEET/CORT/DFP model, the GWI group started out with significantly smaller cerebellar volumes compared to control. This reduction is in line with clinical observations of cerebellar atrophy in GWI veterans and indicate that this model’s chemicals may contribute to this pathology (Christova et al., 2017). Age-associated declines in volume were also apparent for the pons and medulla within both models, albeit not significant for the PB/PM model. These results are consistent with findings in veterans with GWI (James et al., 2017; Zhang et al., 2020; Zhang et al., 2021). Like the cerebellum, both areas of the brainstem were smaller across the time points examined in a model-specific manner. Here, the GWI group of the PB/DEET/CORT/DFP model had smaller pons and medulla volumes compared to control, as early as 6–9 months. As the brainstem is an important regulatory area for vital functions such as respiration and sleep (Moini and Piran, 2020), further characterization of these functions within these models and in veterans with GWI is warranted.

Overall, this study provides insight into the progressive impact of GWI chemical exposures on brain structures that may be associated with previously reported behavioral and neurological effects observed in these models (Zakirova et al., 2016; Brown et al., 2021a; Carpenter et al., 2021). The data obtained here provide a comprehensive summary of the impact of these models’ chemical exposures, especially when considered alongside our earlier studies, which identified significant behavioral impairments, neuroinflammation, and aberrations in synaptic transmission, particularly at the 9-month time point (Brown et al., 2021a; Brown et al., 2021b; Carpenter et al., 2021; Carpenter et al., 2022). Structural alterations we observed here, especially the markedly earlier total brain volume reduction in PB/DEET/CORT/DFP model are indicative of accelerated brain aging. This is in line with multiple recent reports where (1) the rate of mild cognitive impairment (MCI) was greater in veterans with GWI in two studies (Chao, 2020; Chao et al., 2023), (2) Gulf War veterans not only have greater rates of MCI, but also experience olfactory impairments (Chao, 2024), and (3) veterans with severe GWI symptoms might be impacted by accelerated aging the most (Petry et al., 2024; Thompson et al., 2024). GWI is heterogeneous among veterans; this heterogeneity is attributed not only to varying symptom presentation and severity, but also to diverse chemical exposures during the Gulf War, including exposure to sarin/cyclosarin among a subset of individuals (White et al., 2016). This study provides valuable insights into the structural differences between distinct chemical exposures, such as the earlier PB/PM hippocampal and PB/DEET/CORT/DFP brainstem deficits that may benefit future investigations into targeted neuroprotective interventions based on GWI exposure and symptom endophenotypes.

## Data Availability

The raw data supporting the conclusions of this article will be made available by the authors, without undue reservation.

## Footnotes

^1^ https://mouse.brain-map.org/static/atlas
