# Supplementary material for: Longitudinal evaluation of structural brain alterations in two established mouse models of Gulf War Illness
Source: Front Neurosci. 2024 Sep 6;18:1465701. doi: 10.3389/fnins.2024.1465701 (PMC11412963; doi:10.3389/fnins.2024.1465701)
Supplement: Supplementary file 1 [file Table_1.DOCX]

| **Supplemental Table 1. Percent Volume Change from 6 Months to End of Study in Both Models of Gulf War Illness** | | | | |
| --- | --- | --- | --- | --- |
| **Brain Region** | **PB/PM Model** | | **PB/DEET/CORT/DFP Model** | |
|  | Control | PB/PM | Control | PB/DEET/CORT/DFP |
| Total Brain | -0.21 | 0.42 | -0.08 | -0.96 |
| Ventricles | 17.30 | 25.44 | 11.02 | 20.22 |
| Hindbrain | 0.21 | -3.95 | -2.15 | -1.27 |
| Cortex | -0.09 | -0.24 | -0.56 | -2.00 |
| Hippocampus | -7.75 | -11.40 | -5.03 | -2.03 |

**Supplemental Table 1. Percent Volume Change from 6 Months to End of Study in Both Models of Gulf War Illness.**  The percent change in volume from 6 months to 12 months was calculated for the PB/PM and PB/DEET/CORT/DFP models of Gulf War Illness. Positive values indicate an increase in volume, while negative values indicate a decrease in volume. Abbreviations: CORT, corticosterone; DEET, N,N-diethyl-meta-toluamide; DFP, diisopropylfluorophosphate; PB, pyridostigmine bromide; PM, permethrin.
